# Heading direction with respect to a reference point modulates place-cell activity

**DOI:** 10.1038/s41467-019-10139-7

**Published:** 2019-05-27

**Authors:** P. E. Jercog, Y. Ahmadian, C. Woodruff, R. Deb-Sen, L. F. Abbott, E. R. Kandel

**Affiliations:** 1 Department of Neuroscience, College of Physicians and Surgeons of Columbia University, New York, NY 10032 USA; 2 Institute of Neuroscience, Department of Biology and Mathematics, University of Oregon, Eugene, OR 97401 USA; 3 Department of Physiology and Cellular Biophysics, College of Physicians and Surgeons of Columbia University, New York, NY 10032 USA; 4 Zuckerman Mind Brain Behavior Institute, Columbia University, New York, NY 10027 USA; 5 Kavli Institute for Brain Science, Columbia University, New York, NY 10032 USA; 6 Department of Psychiatry, College of Physicians and Surgeons of Columbia University, New York, NY 10032 USA; 7 Howard Hughes Medical Institute at Columbia University, New York, NY 10032 USA

**Keywords:** Neuroscience, Cognitive neuroscience, Computational neuroscience, Spatial memory

## Abstract

The tuning of neurons in area CA1 of the hippocampus emerges through a combination of non-spatial input from different sensory modalities and spatial information about the animal’s position and heading direction relative to the spatial enclosure being navigated. The positional modulation of CA1 neuronal responses has been widely studied (e.g. place tuning), but less is known about the modulation of these neurons by heading direction. Here, utilizing electrophysiological recordings from CA1 pyramidal cells in freely moving mice, we report that a majority of neural responses are modulated by the heading-direction of the animal relative to a point within or outside their enclosure that we call a reference point. The finding of heading-direction modulation relative to reference points identifies a novel representation encoded in the neuronal responses of the dorsal hippocampus.

## Abstract (web summary)

Place cells are neurons in the hippocampus which encode an animal’s location in space. Here, in mice, the authors show that place cell activity is also modulated by the heading-direction of the animal relative to a particular “reference point” that can be either within or outside their enclosure.

## Introduction

Successful navigation requires knowledge of both location and direction, quantities that must be computed from self-motion and from environmental cues. Place cells in the dorsal hippocampus^1^ and grid cells in entorhinal cortex^2,3^ are the best known example of neurons modulated by location. In addition, neurons modulated by head direction have been reported in various brain areas^4–9^, including the hippocampus^7,8,10–12^. In some cases, firing rates are modulated by the animal’s spatial relationship to visual cues^8^, to objects^13,^^14^, or to task goals^15^. Here we provide evidence for a novel form of direction tuning of place-selective neurons in area CA1 of the mouse hippocampus: modulation of neurons by heading direction (HD) relative to various reference points located inside or outside the enclosure. These results extend the scope of spatial representations in the hippocampus beyond the well-known place-cell maps.

## Results

### HD modulation of place-cells activity

We recorded the activity of 1244 neurons in the dorsal region of both the left and right hippocampi (area CA1; “Methods”; Supplementary Fig. 1) in 12 C57BL/J6 mice. We recorded daily during 4 sessions of 10 min each over a period of 10 days. The analysis presented here is based on 697 neurons that satisfied a set of minimal response and sampling criteria (“Methods”). During each session, food-deprived animals foraged freely within a 50-by-50 cm enclosure. In addition to monitoring the location of the animal (*x, y*), we tracked its absolute HD angle (*H*), defined as the angle of the animal’s direction of motion relative to a fixed orientation in the room that coincided with a cue card on one wall of the enclosure (“Methods”). Motion direction was determined by computing the difference between the locations of the animal at two times separated by 1/30 s. For these analyses, the enclosure was divided into 100 square spatial bins (5-by-5 cm each). The heading angle was in turn divided into 10 angular bins of 36° each. To obtain the firing rate data r(x, y, H), firing rates of the recorded neurons were averaged across multiple occupancies within each bin to obtain the firing-rate vector **r**(*x, y, H*).

**Fig. 1:**
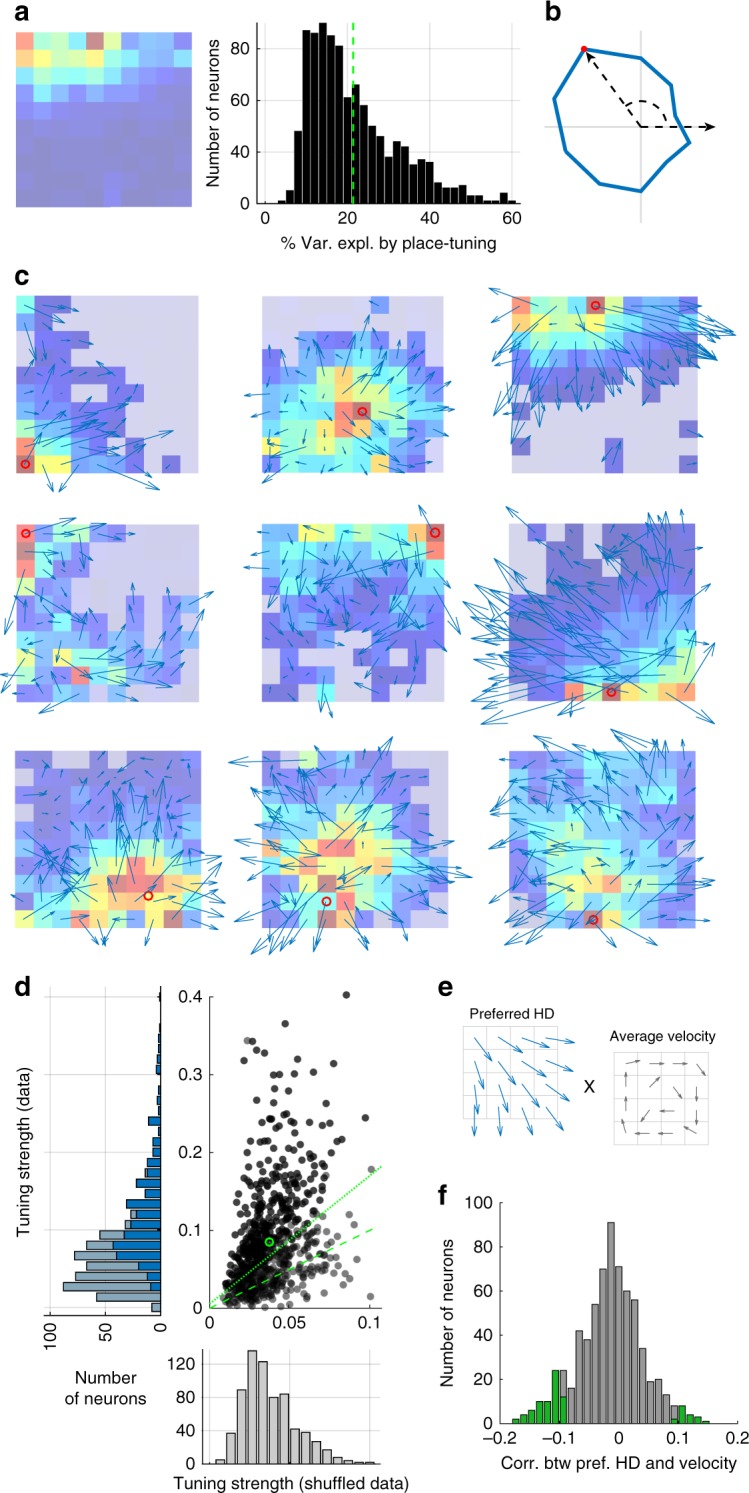
Heading-direction (HD) modulation. **a** Firing rate as a function of location for a typical place cell (left). Firing rate range for spatial map in spikes per second (Hz) is 0–13.8. Colors from blue to red indicate low to high firing rates. (right) Percentage of variance explained by location tuning across neurons. **b** Example of HD tuning in one neuron. Radius of curve is proportional to the firing rate for each angular bin. Maximum firing rate for angular dependency 3.2 (Hz). **c** Head-direction tuning of firing rates shown as rate-weighted sums of HD vectors within each bin. Place fields are also shown, as in a. Firing rate ranges for each spatial map are 0–9.5, 0–5.2, 0–13.8; 0–5.9, 0.1–28.5, 0–8.8; 0.1–10.4, 0–7.8, and 0–8. Red circles are the centers of mass of the place fields. Light-blue squares denote bins with average firing rates too low to be included in the analysis of directional modulation. **d** Average tuning strengths of HD modulation across bins for actual and randomly reshuffled data. Green circle denotes the means of each distribution. Neurons that have average HD modulation higher than the 95% confidence of the shuffled data angular modulation are above the dotted green line (dashed green line is the diagonal); 78% of the neurons are above the equality line (dashed line), and 46% are above the 95% confidence line (dotted line). Histogram of neurons with tuning strength for all neurons and for neurons above 95% confidence interval in light and dark blue bars, respectively. **e** Schematics of preferred HD vectors (left) and average velocity vectors (right) for each spatial bin. **f** Distribution of the correlation between preferred HDs (pref. HD) and the velocity of the animal’s motion for actual and shuffled data. Only 13.4% of neurons, indicated by green, have significant correlation between neural response and direction of motion (5% is expected)

Responses of most of the recorded neurons were modulated by the location of the animal (Fig. 1a, left), and 69.5% had significant spatial information based on a standard shuffling procedure (95th percentile significance threshold; “Methods”; we did not restrict our general analysis to these neurons). This percentage is similar to that recently reported in bats using the same method (69% in ref. ^15^). Even for the most significant neurons, place tuning accounts for a relatively small percentage of the variance of the recorded activity (typically <50% and often considerably less; Fig. 1a, right), so we investigated other sources of firing-rate modulation. In particular, we examined the effect of HD on neural activity (Fig. 1b).

We obtained a conventional place-cell description from our data by averaging the rates **r**(*x, y, H*) over heading angles to yield a place-modulated rate **r**(*x, y*). To examine directional modulation independent of spatial modulation, we considered the ratio **R**(*x, y, H*) = **r**(*x, y, H*)/**r**(*x, y*). To avoid dividing by zero or near zero rates, we restricted this computation to spatial bins with **r**(*x, y*) > 0.5 Hz (results did not change when we modified this cutoff). We also imposed a requirement that each spatial and angular bin used to compute **R**(*x, y, H*) was occupied for at least 500 ms, on at least 4 separate occasions, to avoid under-sampling (although the average number of passes through each spatial bin was 72).

To look for modulation by HD, we defined 10 unit vectors for each spatial bin pointing in the directions defined by the centers of the 10 angular bins. We then weighted these vectors by **R**(*x, y, H*) and summed over *H* to determine a preferred heading vector for each spatial bin (Fig. 1c; Supplementary Fig. 2). We quantified overall HD tuning by computing the lengths of these preferred heading vectors and averaging them over all the spatial bins to obtain a tuning strength for each neuron across the entire environment. We then compared these tuning strengths with results obtained from surrogate data created by randomly shuffling heading angles across the entire session (“Methods”). In this analysis, 46% of the recorded neurons had statistically significant (outside the 95% confidence interval) HD modulation (Fig. 1d). In addition, 78% of the cells had HD modulation greater than the average for the shuffled data (significant population effect *χ*^2^ test: *p* « 0.001).

We next tested whether the HD dependence arose from a lack of uniformity in angular sampling due to the behavior of the mice^12^. To the contrary, we found that omitting spatial bins with poor angular sampling or biased distributions of heading angles from the analysis actually increases HD tuning (Supplementary Fig. 3). Our shuffling procedure did not preserve the continuity of HD across time. We therefore created alternative surrogate data sets by shifting the heading angles in time relative to the spatial location of the animal (“Methods”). This preserved continuity, but when the temporal shift is large enough, it decouples *H* from the actually spatial location where it was measured. The HD tuning in these surrogate data decreases when the temporal shift is greater than a few hundred milliseconds (Supplementary Fig. 4), demonstrating that the tuning is sensitive to the actual relationship between HD angle and location. We also tested whether within a session HD tuning is sensitive to an animal’s overall direction of motion or velocity. For each neuron, we computed the dot product between the preferred HDs for each spatial bin and the average velocity taken by the animal through that bin, summing over all spatial bins (Fig. 1e). The resulting distribution of correlation values was very similar to the distribution we obtained when the trajectories were randomly shuffled, with only 13.4% of neurons being significantly different from shuffle (outside the 95% confidence interval, so 5% would be expected; Supplementary Fig. 5). We did an additional analysis for the correlation of HD with the animal’s direction of motion and obtained similar results (11.2% of cells significantly different from shuffle; Fig. 1f). Finally, we illustrate in the middle row of Fig. 1c an example of three neurons that were recorded simultaneously and thus have identical behavioral effects but distinct HD neural responses and different preferred directional tunings.

### Model of HD modulation relative to a reference point

**Fig. 2:**
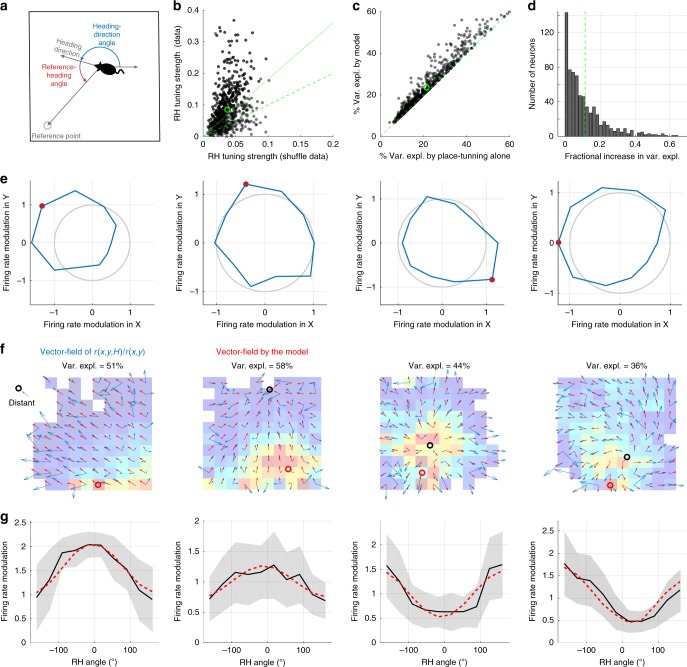
Model of modulation by heading direction relative to a reference point. **a** Definitions of the reference point and reference-heading (RH) angle. **b** RH-angle tuning for the recorded population of neurons compared to the tuning for surrogate data with randomly shuffled heading directions. Green circle is defined as in Fig. 1. 86% of the neurons are above the equality line (dashed line), and 56% are above the 95% confidence line (dotted line). **c** Variance explained by the model with both RH angle and place modulation compared to a purely spatial description. **d** Histogram of the fractional change in variance, defined as the change in variance due to RH modulation divided by the variance explained solely by place modulation. **e** Heading-direction tuning with spatial tuning removed over the full range of ±180 degrees divided into 10 bins. The eccentricity (around the circle of mean rate) of the polar plot represents the heading-direction modulation of the firing rate. **f** Same example cells as in **e**, showing spatial tuning (heat map), heading-direction tuning (blue arrows), and RH-angle tuning as fitted by the model (red arrows). Black circles are the reference points obtained by the model. Reference point far from the arena enclosure is noted by an arrow with the word “distant” pointing toward the reference point. **g** RH-angle tuning responses fitted by the model. Shaded area is the ±std.dev. These tunings are comparable with reports in bats^15^

Having demonstrated HD tuning in general, we proceeded to characterize the structure of this tuning across the spatial extent of the enclosure. To construct a more general model than simple modulation by heading relative to a fixed direction in the room and inspired by the recent work of Sarel et al.^15^, we considered firing-rate modulation as a function of the angle (*θ*) between the HD and a line extending from the animal to a fixed location in the room, either within or outside the enclosure (Fig. 2a). We called this fixed location, determined by fitting, the reference points and the angle thus defined, the reference-heading (RH) angle. Specifically, we considered the model **R**(*x, y, H*) = *α* *+* g*cos(*θ* − *θ*_p_), where we fit 4 parameters for each neuron: *g*, the modulation strength; *θ*_p_, the preferred RH angle; and 2 parameters describing the coordinates of the reference point. The angle *θ* depends on *x, y, H* as well as on the location of the reference point. The value of *α* is determined not by fitting but by imposing the fact that, by construction, the average of **R**(*x, y, H*) over HD angle *H* is 1 (“Methods”). Thus *α* values different from 1 correct for sampling biases in *θ*.

After fitting the model and obtaining the best-fit reference points, we defined the overall RH tuning strengths for each neuron using the same vector summing and length averaging that we applied to compute heading-angle tuning strengths (Fig. 1d). We then compared the results with the shuffled data. Most of the neurons (86%) showed greater RH tuning strength for actual than for shuffled data, and 56% show a significant tuning (outside the 95% confidence interval; Fig. 2b). We also computed the percentage of accounted variance for rates modulated by the fitted model and compared them with the accounted percentage using only position-modulated rates (Fig. 2c). The RH modulation of the model increased the variance explained for the recorded neurons by up to a factor of 0.6, with an average fractional increase of 0.12 (Fig. 2d). Figure 2e–g show examples of neuronal activity fitted by the model (these are the same neurons as shown in the last four examples in Fig. 1c; see also Supplementary Fig. 6). We found similar percentages when we performed fits of data from two other environments (see “Methods” section for description of the other two environments). Thus RH modulation does not depend on a particular environment but, instead, appears to be a general property.

### Distribution of reference points relative to the spatial enclosure

**Fig. 3:**
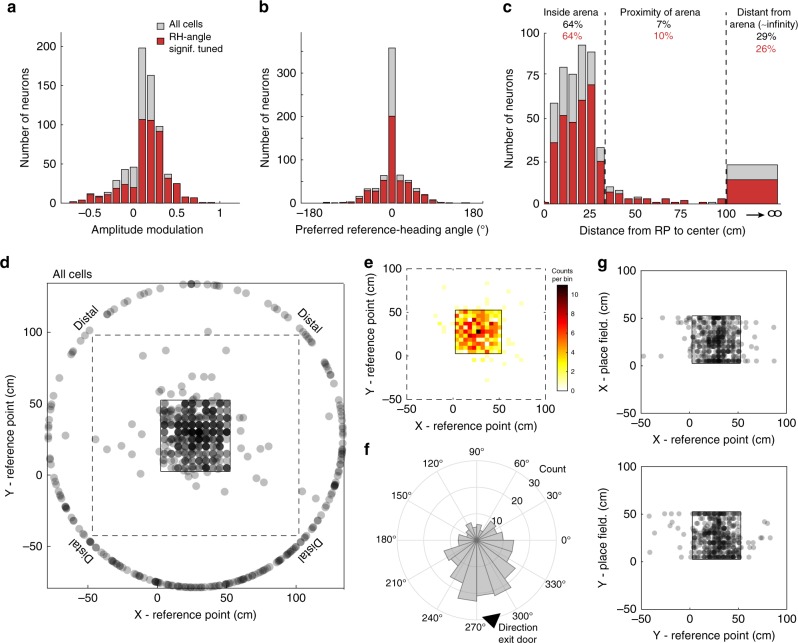
Fitted model parameters across neurons. **a** Histogram of the amplitude of the angular modulation for all neurons and for neurons with significant reference-heading (RH)-angle tuning (neurons above the dotted line in Fig. 2b). **b** Histogram of the angle of the preferred RH direction. **c** Histogram of the distances of reference points from the center of the enclosure. **d** Reference points located within the enclosure (square in the center of the panel), in the proximity of the enclosure (dashed square), and at distal locations (collapsed onto a circle of 150 cm diameter). **e** Density map of reference point locations inside and near the enclosure. The concentration of reference points toward the center of the arena is not statistically significant. **f** Circular histogram of the direction to reference points at distal locations shows a significant concentration in the S-E direction (outside the 95% conf. interval). reference points within the box do not show any significant anisotropy. **g** Scatter plot of reference point coordinates (*x*—top, *y*—bottom) compared to the coordinates of the centers of the place fields for each neuron. There is no correlation between the locations of the reference points and the place field center of mass for the same neuron

The fitting process generates distributions of modulation strengths (*g*; Fig. 3a) and preferred RH angles (*θ*_p_; Fig. 3b), as well as the coordinates of the reference points (Fig. 3c). Preferred RH angles are highly peaked around zero (Fig. 3b), which means that the maximum modulation occurs when the animal is heading toward the reference point. Most, but not all, of the modulation strengths are positive, so that the rate is maximized when the animal is heading toward the reference point. However, the modulation strengths for 38% of the neurons are negative (Fig. 3a), which means in this case that the rate is maximally suppressed. Reference points for 64% of the neurons were inside the enclosure, 7% are outside but near the enclosure, and 29% were distal (Fig. 3c; Supplementary Fig. 7). Model parameters for cells marked as statistically significant in Fig. 1d or 2b showed the same ranges and distributions as for all other neurons, so we combine results from all of the fitted neurons.

Because our model reduces to a pure HD tuning when the reference point is far from the animal (Supplementary Fig. 8), neurons with distal reference points correspond to head-direction modulated neurons. The distal reference points we extracted show an angular preference (Fig. 3d–f), with the density peaking at a direction that coincides with the exit door of the experimental room, which is almost due south on the schematics (Fig. 3f and Supplementary Fig. 9). In contrast to the distal reference points, the distribution of proximal reference points that are within the enclosure, corresponding to distinctively RH-modulated neurons, do not show any angular preference, but they have a higher density near the center of the arena than near its edges. This difference does not reach statistical significance, however (Fig. 3d, e). We also found no correlation between the location of the reference point within the enclosure for a given neuron and the center of its place field (Pearson’s correlation coefficient *r* = −0.05 for *x* coordinate and *r* = −0.03 for *y* coordinate; Fig. 3g; “Methods”). This implies that place and RH tuning use two different reference systems.

## Discussion

We have found that the firing rates of the majority of neurons in the CA1 region of the dorsal hippocampus are modulated by the HD of the animal relative to a reference point in the environment. Most of the neurons have a higher response when the animal moves toward the location of the reference point, as was also found by Sarel et al.^15^ In their analysis of goal-location cells, Sarel et al.^15^ identified a separatrix between the subset of cells that were better explained by a place model and those that were better explained as goal-selective. We do not interpret RH-modulated neurons as a subtype of hippocampal neuron. Instead, we propose an additional spatial dependence for hippocampal activity that appears, with varying levels of modulation, across the full population of responsive neurons.

Previous work has found that neurons in area CA1 of the dorsal hippocampus are modulated by HD relative to a specific orientation, object, or relationship to visual cues^6–8,10,15^. We monitored heading rather than head direction, in other words, the direction of movement of the animal. Although head and HD are often the same while animals are moving, differences have been reported in the relationship of the two with neural activity^16^. For reference points located far from the enclosure, our model reproduces previously reported head direction-tuned responses in the hippocampus^7,8,10–12,15^ and tuning with respect to a specific landmark, namely, the door of the experimental room.

Previous work on the modulation of activity with respect to HD relative to location in space considered locations that were distinguished by the presence of an object or a visual cue^6,9,13,14^ or a reward^15^. Our work reveals modulation of place-cell activity by HD relative to unmarked points in the environment; reference points inside the enclosure seem to have no particularly distinguishing characteristic. RH tuning may be related to the type of visual-cue modulation reported by Wilber et al.^6^ and Acharya et al.^8^. It would be interesting to use our model and approach to analyze other data sets that identified object- or goal-direction tuning to see whether more general reference-point tuning is also present in these data. Conversely, our analysis could be extended to include objects and goal locations in the environment.

What are the origins and functional significance of modulation with respect to RH angle? Determination of spatial location requires integration of a large number of factors, including both proximal and distal sensory cues and internal measures of path integration. Because these factors are key components of the position signal that drives place-selectivity in the hippocampus, it is not surprising to see them reflected in the modulation of place-cell activity. Furthermore, these factors are likely to be correlated with both well-defined and subtly defined features and locations in the environment. Thus any model that takes into account the complexity of location-determining computations would be expected to include modulation by factors that are more complex than pure location tuning. The modulation by HD with respect to unmarked reference points that we report matches what would be expected from such a model.

## Methods

### Subjects

Data were recorded from 12 male C57BL/6J mice. In 4 mice, 1 microdrive with a bundle of 4 tetrodes was implanted in the right hippocampal area CA1, and the other 8 mice were implanted with two microdrives each, with 4 tetrodes in each hemispheres (area CA1: 1.8 mm DL, 1.8 mm RC, ~1.1 mm deep). Mice were 4–6-month old (30–34 g) at the time of the implantation surgery. After surgery, all mice were individually housed under 12 h light/dark cycle and provided water ad libitum and one food pellet overnight (maintaining the same body weight as prior to the implantation (+the implant)). All housing, surgical, and behavioral procedures conformed to National Institute of Health (NIH) standards using protocols approved by the Institutional Animal Care and Use Committee (IACUC).

### Electrode implantation and surgery

Custom-made, reusable microdrives (Axona) were constructed by attaching an inner (23 ga) stainless steel cannula to the microdrive’s movable part to allow inserting the electrode reliably in 10-micron steps. An outer (19 ga) cannula covered the exposed tetrode section from the dura to the inner cannula. Tetrodes were built by twisting four 17-μm-thick platinum–iridium wires (California wires) and then heat bonding them. Four such tetrodes were inserted into the inner cannula of the microdrive and connected to the wires of the microdrive. Prior to surgery, the tetrodes were cut to an appropriate length and plated with a platinum/gold solution with additives^17^ until the impedance dropped to 80–100 KΩ. All surgical procedures were performed following NIH guidelines in accordance with IACUC protocols. Mice were anesthetized with a mixture of 0.11 ml of Ketamine and Xylazine (100 and 15 mg ml^−1^, respectively) per 10 g body weight. Once under anesthesia, mice were fixed to the stereotaxic unit with head restrained using ear-bars. The head was shaved and an incision was made to expose the skull. Ten jeweler’s screws were inserted into the skull to support the microdrive implant. Two of these screws were soldered to wires and screwed until they pierced the dura, which served as a ground/reference for electroencephalogram recordings (one in olfactory bulb and one in the cerebellum). A 2-mm hole was made in the skull at position 1.8 mm medio-lateral and 1.8 mm antero-posterior to the bregma junction. Tetrodes were lowered to about 0.7 mm from the surface of the brain (dura). Dental cement was spread across the exposed skull and secured to the microdrive. Any loose skin was sutured back in place to cover the wound. Mice were given Carprofen (5 mg kg^−1^) post-operatively to reduce post-procedure pain. Mice usually recovered within a day after the tetrodes were lowered.

### Histology

To determine the exact final position of the tetrodes in a given animal, we performed Nissl dye staining on hippocampal slices. After the experiment was concluded, mice were anesthetized with an overdose of 0.5 ml Ketamine and Xylazine solution (100 and 15 mg ml^−1^, respectively) and intra-cardiac perfused with 40 ml of 4% paraformaldehyde (PFA) solution, immediately after the tetrodes were retracted and before mice were decapitated. The skull was cut open from the bottom of the head to expose the brain, which was gently removed and stored in 4% PFA solution for 24 h in a shaker. The brain was coronally sliced in 30-μm-thick sections using a cryostat at −13 °C. The sections were stained with fluorescent Nissl dye (Neurotrace) and mounted onto a slide. The brain sections were viewed under a high-magnification microscope, and digital pictures of the slices were taken (Supplementary Fig. 1).

### Experimental methods

After animals recovered their normal weight a few days post-surgery, the electrodes were connected to an amplifier to detect single cells. Tetrodes were lowered ~12.5 microns twice a day (6 h apart) until the number of recorded cells was maximized (this process took between 7 and 10 days). After the depth of the electrode was optimal, we waited 3–8 days for recording stabilization. Animals were food deprived for 4 h before each recording session over the experimental period of 7–10 days. Animal’s were reconnected to 16 or 32 channels for every single session of 10 min and introduced in the same region of the arena relative to the recording room in each of the three environments. Each environment is approximately 50 cm wide with walls that are 40 cm in height. All three environments present a cue card on the internal side of the enclosure with the same orientation relative to the room for all three environments (north on our plots in Fig. 1) to act as a reference direction for navigation. Environment A was a square enclosure with black walls and white floor that had ground food (vanilla cookie) spread over the surface of the floor and also a small pellet ~10 cm away from the S-E corner of the environment. Environment B was a circular enclosure with black walls and brown textured floor with ground food (cocoa flavored puffed rice) spread over the surface. Environment C was a circular enclosure with white walls and floor and no food. We used these differences in shape and reward distribution to enhance the animal’s ability to differentiate between the environments. Animals were released to freely explore the environments and eat the food. For this paper, we used the data from Environment A, unless otherwise specified.

Recordings were done with Neuralynx amplifiers: Lynx-8, with a total of 16–32 channels depending on the success of the electrode’s implantation surgery. Data were collected in continuous format at a 30 kHz sampling rate and then pre-processed using NDManager^18^. The system records the position of the animal by tracking the location of two leads of different color mounted on the pre-amplifier connector to the recording head-stage on the head of the mouse. High and low frequencies were separated and spike wave-forms were extracted for cell identification. Cell clusters were computed using the EM algorithm implemented in the KlustaKwik software^19^ over the concatenated data of the 12 sessions within a day for each animal. Human inspection over each cluster was done using the Klusters software^18^. Spike times and animal trajectories were transformed into Matlab objects and data analysis was performed using this platform.

By lowering the electrode, we identified the fast spiking neurons around 1000 microns deep from the dura, and then between 50 and 100 microns deeper we found the pyramidal layer. If we move further down (another 50 microns), the activity completely vanished, until reaching dentate gyrus perhaps a full 1 mm below. Thus, consistent in all animals, the location of the recorded layer was approximately 1050–1150 microns from dura, as shown in Supplementary Fig. 1. Any fast spiking neurons remaining after sorting were left out based on their waveform half width and shape of their autocorrelation (long autocorrelations up to seconds).

Before analyzing the data, the experimental days used for the data analysis for a given mouse was determined based on criteria of recording stability. Because we originally wanted to follow the same cells over days (although this is not necessary for the work presented here), we established the following criteria to determine stability: for each cell, we chose the channel with the largest spike-waveform fluctuation over the four channels on a given tetrode. We then computed over that channel the mean waveform in a vector of 40 discretized points (at 30 kHz sampling rate). We calculated the peak amplitude of the mean waveform over all cells and also its standard deviation. We then compared the distributions of peak waveforms for each cell across days using a *t* test for two distributions with different means and standard deviations; when these distributions became statistically different, we stopped using the data from that day on for that particular animal, because this indicated a large movement of the electrode.

### Data analysis

Data pre-processing was performed in the following manner: after units were isolated, cells were considered for the analysis if their average firing rate across the 4 sessions within a day was >0.1 Hz. This threshold left out approximately 2% of the cells. To avoid spikes that were not correlated with mouse position due to pre-play or re-play (e.g., nonspecific spatial firing when animals are not moving within the box)^20,]^^21^, we only kept frames in which the animal was moving faster than 4 cm s^−1^. This threshold was obtained by inspecting behavior movies and selecting by observation a minimum speed that removed frames where animals were not moving their bodies but only their heads. Inclusion criteria for spatial bins was determined when animals spent at least 500 ms in each spatial bin over at least 4 different visits. For each spatial and angular bin (*x*,*y*,*H*, 3-dim, 10 bins per dimension, 1000 total bins), we used spikes only from times that satisfied our threshold criteria. We then computed the mean rate using the spike counts for the time samples from each visit of the animal to a given spatial and angular bin, and we divided the mean spike count by the sampling rate Δ*t* = 1/30 s. In addition to HD (*H*), which we defined using the same definition of HD as Sarel et al.^15^, we also monitored the head direction of the animal measured by the positions of two light-emitting diodes attached to the pre-amplifier connected to the mouse tetrode boards, but this observable was noisier than the HD (*H*) due to the many degrees of freedom of the animal’s head while moving during the session, and we did not use it in our analysis.

The classification of place cells is conducted by the following procedure: cells included in this analysis have >100 spikes per 10-min session (400 spikes for the full 40-min session). We computed the spatial information (SI, in units of bits per spike) of the firing-rate map as

1 $${\mathrm{Spatial}}\;{\mathrm{information}} = \mathop {\sum}\limits_{x,y}^{N{\mathrm{bins}}} {{\mathbf{p}}(x,y) \times ({\mathbf{r}}(x,y)/{\bar{\mathbf{r}}}) \times {\mathrm{{log}}}_2({\mathbf{r}}(x,y) / {\bar{\mathbf{r}}})}$$

where **r**(*x*,*y*) is the firing rate of the cell in the *x*,*y* bin, **p**(*x*,*y*) is the probability of the mouse to be in this spatial bin, and ${\bar{\mathbf{r}}}$ is the mean firing rate of the cell^22^.

A cell was classified as a place cell if its spatial information was statistically significant above the shuffled data (*p* < 0.05), based on the following criteria: spike sequences were time-shifted by a random time interval in a circular manner with the end of the session wrapped into the beginning, to keep the same number of spikes per session. This procedure decouples the spike times from the animal trajectory but preserves the spiking patterns. This shuffle procedure was repeated 100 times for each neuron. A neuron was defined as significantly tuned if the spatial information exceeded the 95th percentile of the shuffled distribution for that session.

Here we describe the shuffling procedures we used to test the statistical significance of the observed results. (1) We created surrogate data in which the HD for the animal was shifted in time relative to the location and firing rate (used for Supplementary Fig. 4). We shifted in a circular manner with the end of the session wrapped to the beginning. (2) We created surrogate data with the same general statistics as the raw data by randomly interchanging the indices of the HD angles at each time (drawing without replacement) but preserving the original positions and firing rates for individual cells. By repeating this procedure a large number of times (*N* = 1000), we obtained 95% confidence interval limits for the surrogated HD tuning curves, as well as a mean surrogated HD tuning curve by averaging. For each neuron, we tested whether HD was higher than for the mean surrogate tuning curve (points above the dashed line in Figs. 1d and 2e; 77% for HD and 86% for RH-angle-modulated cells). We also tested if tuning was higher than the 95% confidence interval limit to define neurons as significantly tuned (46% of HD and 56% of RH-angle-modulated cells). (3) With the same shuffle procedure, we tested whether the preferred HD maps (Fig. 1e and Supplementary Fig. 2) were correlated with the preferred motion direction maps and preferred velocity direction maps, testing whether the tuning response was due to biased animal’s behavior (used in Fig. 1f and Supplementary Fig. 5). We computed the correlation of the individual maps for each spatial bin independently and then averaged over bins to obtain a total correlation per neuron. Correlations between the maps were distributed around zero, for the actual data as well as the shuffled data. For shuffled HD maps, we computed the confidence interval for the correlations and determined which neurons had significantly high correlation (97.5 percentile < correlation or correlation < 2.5 percentile) between neural response and direction of motion (11.2%) and velocity direction (13.4%) (green bars on Supplementary Fig. 5d, e). Because these proportions were low in comparison with the expected correlated neurons (5%), we did not exclude neurons from our analysis. (4) We tested whether the distribution of reference points was clustered in the center of the behavioral box (Fig. 3e). We generated surrogated density maps of reference points (with the same number as for the original map) randomly placed with the limits of the box. We smoothed the original and the surrogated maps with a Gaussian kernel with a 2-bin half width (10 cm). We created a confidence interval for the value of the surrogated map for each spatial bin within the enclosure and tested whether the original density map was above the 95% confidence limit in any of the bins. We did not find any point within the box with a statistically significant difference in density of reference points. (5) To test whether circular histograms of reference point angular positions within the environment and at distal locations have a statistical significant peak, we generated surrogated positions of reference points by randomly generating reference points within and outside the enclosure with the same distribution as the original data. We then created for each set of surrogated reference points a circular histogram of angles of reference point locations within the box and the same for distal locations (>150 cm). For all histograms, we computed a confidence interval (2.5–97.5 percentile) and tested whether the original circular histogram fell outside the interval. We did not find significant directionality for reference points within the box, but we did find significant directionality for points at distal locations (paired sample *t* test: *p* < 0.05), as indicated in Fig. 3f and Supplementary Fig. 8a, b. (6) We tested whether there was a relationship between the location of the reference points and the center of mass of the place fields for each neuron (Fig. 3g). We computed the Pearson's correlation between the reference point and place field center of mass locations for each coordinate independently. Correlations were around zero with no significant *p* value (Pearson’s correlation coefficient *r* = −0.05, *p*_val_ = 0.27 for *x* coordinate, and *r* = −0.03, *p*_val_ = 0.52 for *y* coordinate). We also generated surrogated reference point locations by randomly shuffling the neuron index between reference point and place field center of mass and computed the correlations between the distributions several times. The confidence interval [2.5–97.5%] display a range of correlations between −0.13 and 0.13. Actual data correlations were inside the confidence interval and thus could not be considered significantly correlated, failing to reject the null hypothesis.

### Model fitting

For model fitting, we used neurons that satisfied the following conditions: minimum number of spatial bins that satisfy the acceptance criteria given above is equal to 20 and minimum range of HD angles to which the cell is responsive is more than 50 deg. We also reproduced the same results with lower thresholds of minimum firing rate (0.1 Hz) than the standard 0.5 Hz cutoff.

The model has a periodic dependence on the angle between the HD of the animal’s trajectory and the location of the reference point (see Fig. 1b)

2 $${\mathbf{R}}_{{\mathrm{model}}}\left( {x,y,H} \right) = 1 + g\left( {F - \underline F } \right),{\mathrm{where}}\,F = \cos \left( {\theta - \theta _{\mathrm{p}}} \right)\,{\mathrm{and}}\,\underline F = E\left[ F \right]_H$$

where $$\theta = \arctan \left( {\left( {Y_{{\mathrm{Ref}}.{\mathrm{Point}}} - y} \right)/\left( {X_{{\mathrm{Ref}}.{\mathrm{Point}}} - x} \right)} \right){\mathrm{,}}\,{\mathrm{and}}\;{\mathrm{imposing}}\;{\mathbf{R}}_{{\mathrm{model}}}\left( {x,y,H} \right) \, > \, 0$$.

We fitted the values of *g*, *θ*_p_, *X*_Ref.Point_, and *Y*_Ref.Point_ by minimizing the squared difference between the model and the data, summed over all bins. We performed the minimization using an unconstrained nonlinear optimization procedure, the fminsearch function from MATLAB. For the initial parameter estimates, we used: *g* = 0;*θ*_p_ = 0; *X*_Ref.Point_ = Place-cell’s center of mass *X* coordinate, *Y*_Ref.Point_ = Place-cell’s center of mass *Y* coordinate.

### Statistical test for modeled data

In Fig. 1a, we computed the variance explained by a place description to describe how well the mean rate over all angular bins explain the neural activity,

3 $${\mathrm{Fraction}}\;{\mathrm{variance}}\;{\mathrm{explained}}\;{\mathrm{by}}\;{\mathrm{place}}\;{\mathrm{tuning}} = 1 - \frac{{{\mathrm{Var}}\left[ {r(x,y,H) - r(x,y)} \right]}}{{{\mathrm{Var}}[r(x,y,H)]}}$$

In Fig. 1b, we show, in a polar plot, the average rate for each of the 10 HD angles, averaged over the entire experiment.

For Fig. 1d, we performed a chi-square goodness-of-fit test and rejected the hypothesis that the distributions in the *X* and *Y* axes come from the same distribution with the actual values computed being *p*_value_ = 6 × 10^–14^.

In Fig. 2b, we performed a chi-square goodness-of-fit test and rejected the hypothesis that the distributions in the *X* and *Y* axis were coming from the same distribution with the actual value computed being *p*_value_ = 4 × 10^–15^.

In Fig. 2c, we compared the variance explained by the place tuning to the variance explained by including RH angular dependence where the reference point was fitted by the model and

4 $${\mathrm{Fraction}}\;{\mathrm{variance}}\;{\mathrm{explained}}\;{\mathrm{including}}\;{\mathrm{RH}}\;{\mathrm{tuning}} \\ \ \ = 1 - \frac{{{\mathrm{Var}}\left[ {r(x,y,H) - R_{{\mathrm{model}}}(x,y,H)} \right]}}{{{\mathrm{Var}}[r(x,y,H)]}}.$$

## Supplementary information

Supplementary Information

Source Data

## Data Availability

Data from all figures are provided in supplementary material file called: “SourceData” file. All cells raw data are available at CRCNS - Collaborative Research in Computational Neuroscience (http://crcns.org/data-sets/hc/hc-22).
