# Supplementary material for: Heading direction with respect to a reference point modulates place-cell activity
Source: Nat Commun. 2019 May 27;10:2333. doi: 10.1038/s41467-019-10139-7 (PMC6536526; doi:10.1038/s41467-019-10139-7)
Supplement: Supplementary file 1 — Supplementary Information [file 41467_2019_10139_MOESM1_ESM.pdf]

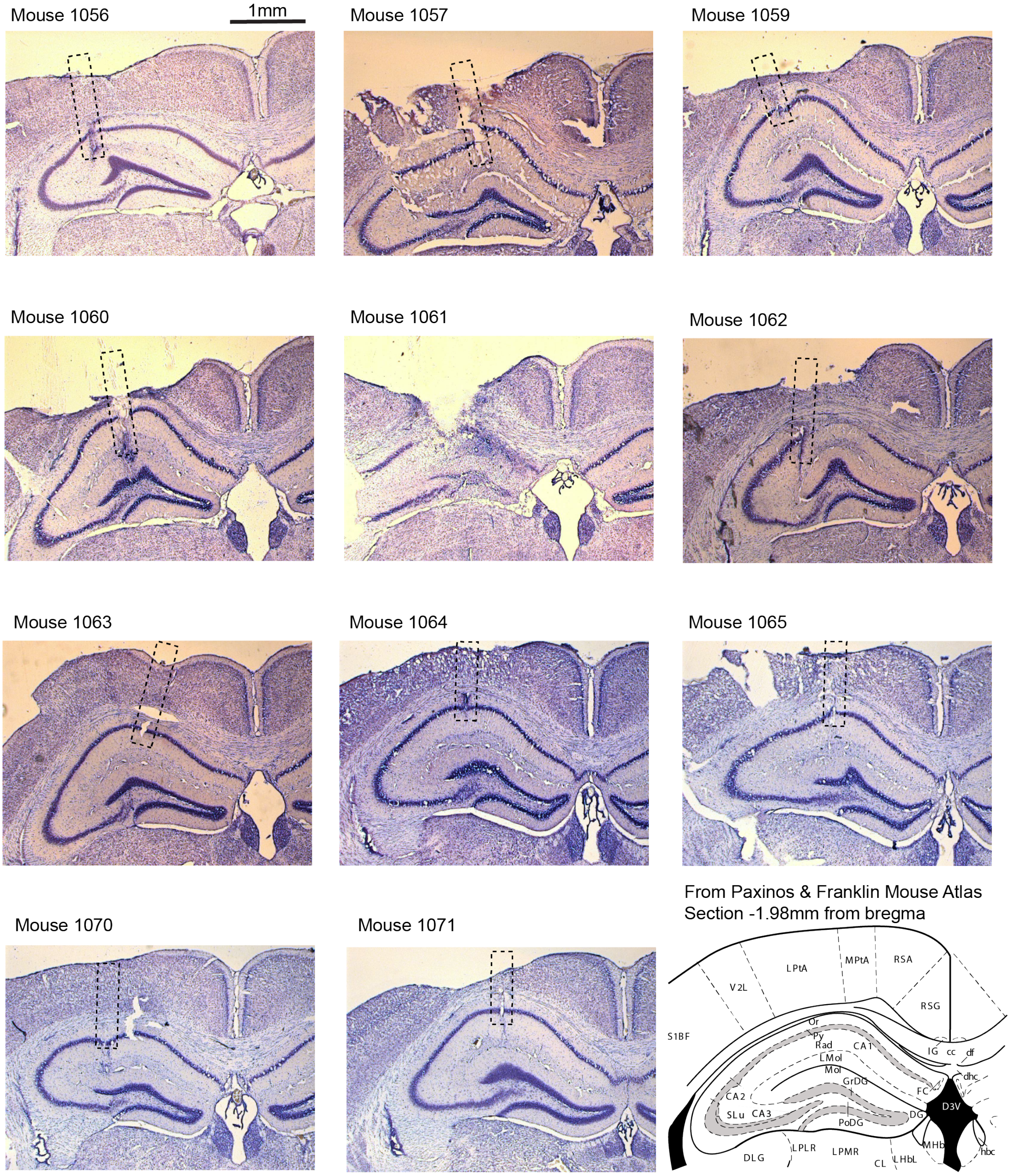

Supplementary Figure 1: Electrode tracks in CA1 region of the mouse hippocampus. Brain section from 11 out the 12 animals used for the electrophysiological recordings (Mouse 1069 is missing). Dashed line rectangles identify the direction of the tetrodes track from the surface of the brain to the CA1 region. Some tracks pass through CA1 because in those animals we also recorded in DG or CA3. Bottom-Right panel shows the section from the mouse atlas for the corresponding brain section of the mouse brain. Tetrodes where implanted at +/-1.8 mm on each hemisphere (medio-lateral), and a -1.8 mm (rostro-caudal) from bregma. The depth of the electrodes when we found the pyramidal layer was between 1050 to 1150 microns from the surface of the brain. **Note:** bottom left figure adapted from (*The Mouse Brain in Stereptactic Coordinates (Second edition)*, Paxinos and Franklyn, Academic Press (1997)).

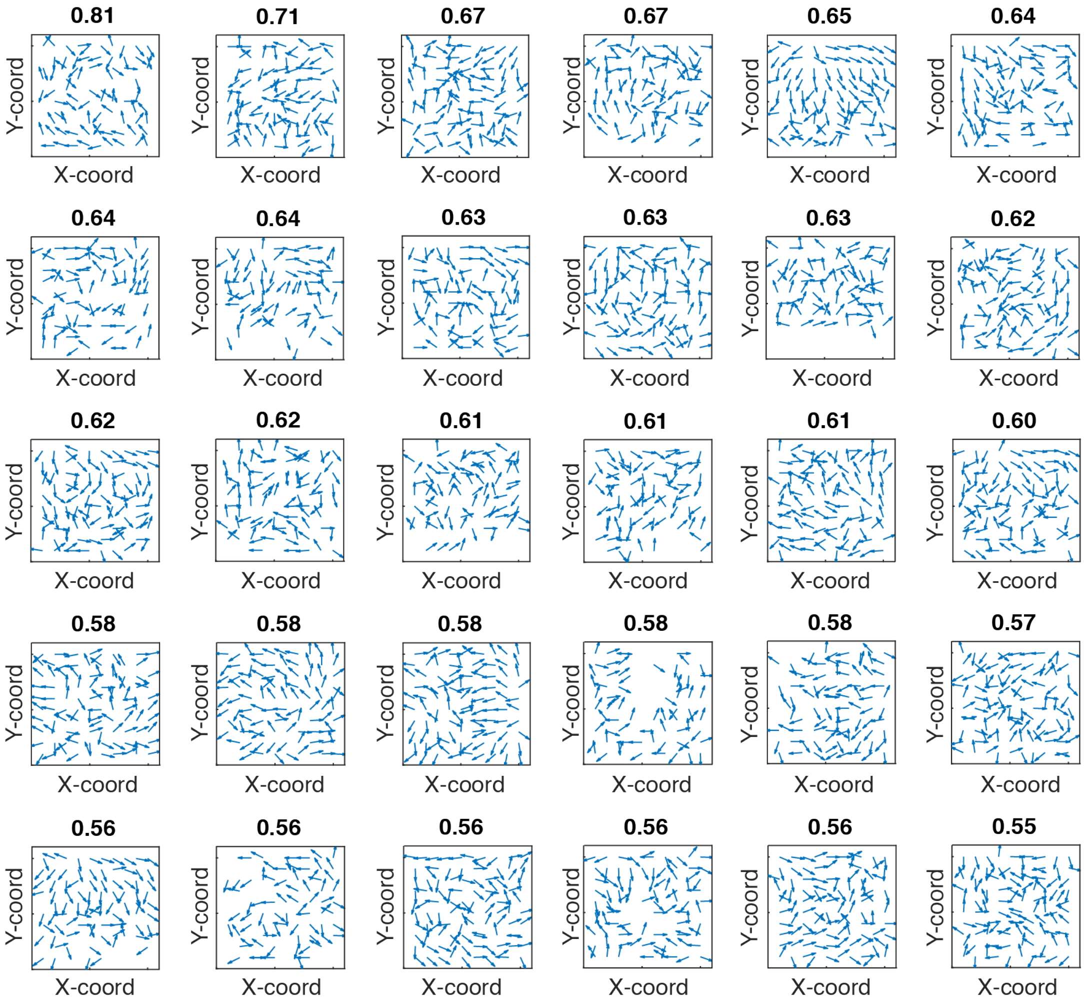

Supplementary Figure 2: Preferred heading-direction response maps. Examples of preferred heading direction vectors for each of the 100 spatial bins. We normalized the vectors to better visualize the organization of prefer heading direction responses over different spatial bins on the map. The structure of the vectors on the maps smoothly changes from bin to bin showing, in some neurons, a globally consistent structure resembling a curl, a divergence or a uniform direction vector field. Number on top of each panel is the highest eigenvalue of the singular value decomposition applied to the correlation matrix between heading-direction tuning vector field and its position on the spatial 2-D bin grid. The higher the number, the smoother is the heading-direction responses across adjacents bins.

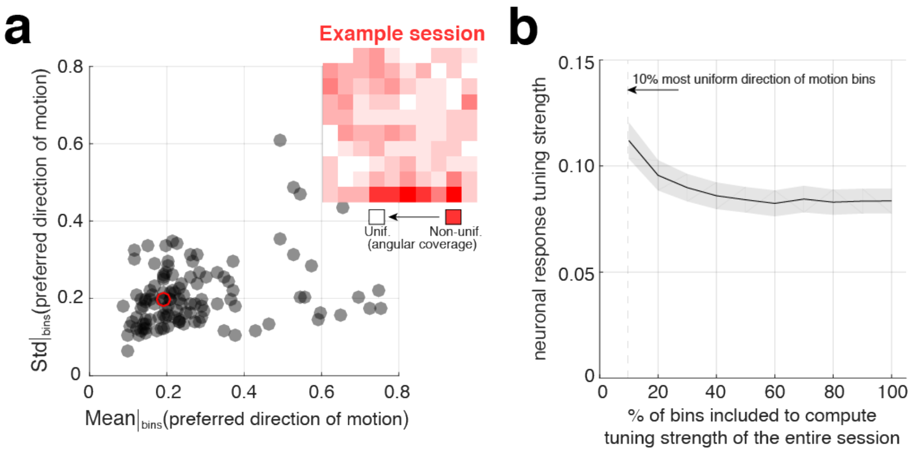

Supplementary Figure 3: Non-uniformities in the direction of motion per spatial bin does not explain the angular tuning of the neural responses. Each spatial bin has a distribution of directions of motion summarizing the directionality of the trajectories when the animal visited that spatial bin. **a)** For some sessions, the angular distribution per bin is not uniform, as represented by the map in red tones at the top-left plot. The more red the less uniform are the visits over a combined session of 40 min. The distribution over the bins for the whole environment is shown by a plot of the mean and std of distributions per bin (black points). Red circle is the "average" sessions represented on the map above. **b)** Mean +/- st.e.m of vector-strength for the populations of neurons when spatial bins with different levels of heading direction coverage are included, starting with the bins having the top 10% uniformity and extending until all of the bins are included, indicated by 100%. Angular tuning decreases when bins with lower uniformity are included in the computation of vector-strength.

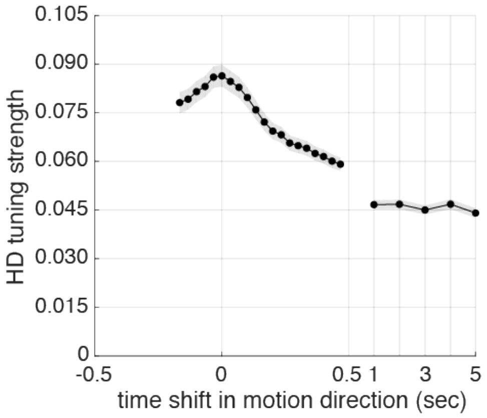

Supplementary Figure 4: Heading direction tuning drops after a time shift of the animal's heading-direction. We rigidly shifted the motion direction angle in time over the entire session by different amounts from -0.15 to +5 s. We left all other values from the data (the mouse position and the firing rate of each individual neurons) unchanged. For each time shift we compute the heading-direction tuning strength of the entire population. Tuning strength of the heading direction decreases for non-zero temporal shifts.

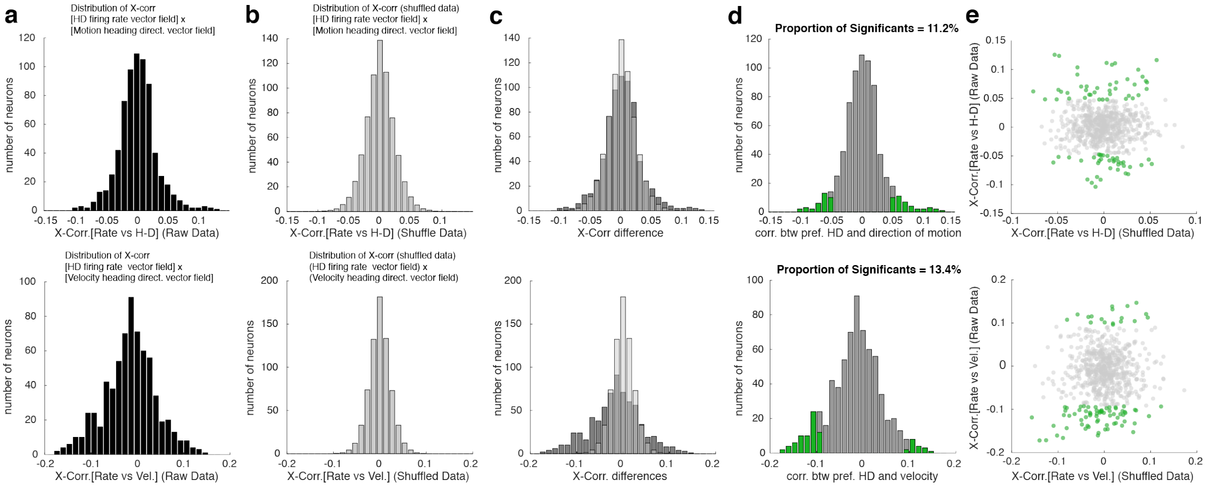

Supplementary Figure 5: HD tuning maps are not correlated with motion direction or velocity direction maps in the majority of recorded neurons. a) correlation between the preferred heading direction (pref. HD) and the average per bin of either the direction of motion (top) or the velocity (bottom) of the animal for the actual data. **b)** Same as in a, but for shuffled data. **c)** Comparison between actual and shuffled data. **d)** Neurons with statistically significant correlation between the preferred heading direction and either the direction of motion (top) or the velocity (bottom) are shown in green bars over the full population (gray bars). Only 11.2% or 13.4% neurons have HD tuning maps significantly correlated with the direction of motion or velocity (the lower panel also appears as Fig. 1f). **e)** Scatter plot of correlation between the preferred heading direction maps and either the direction of motion maps (top) or the velocity direction maps (bottom) for actual versus shuffled data (1000 shuffles).

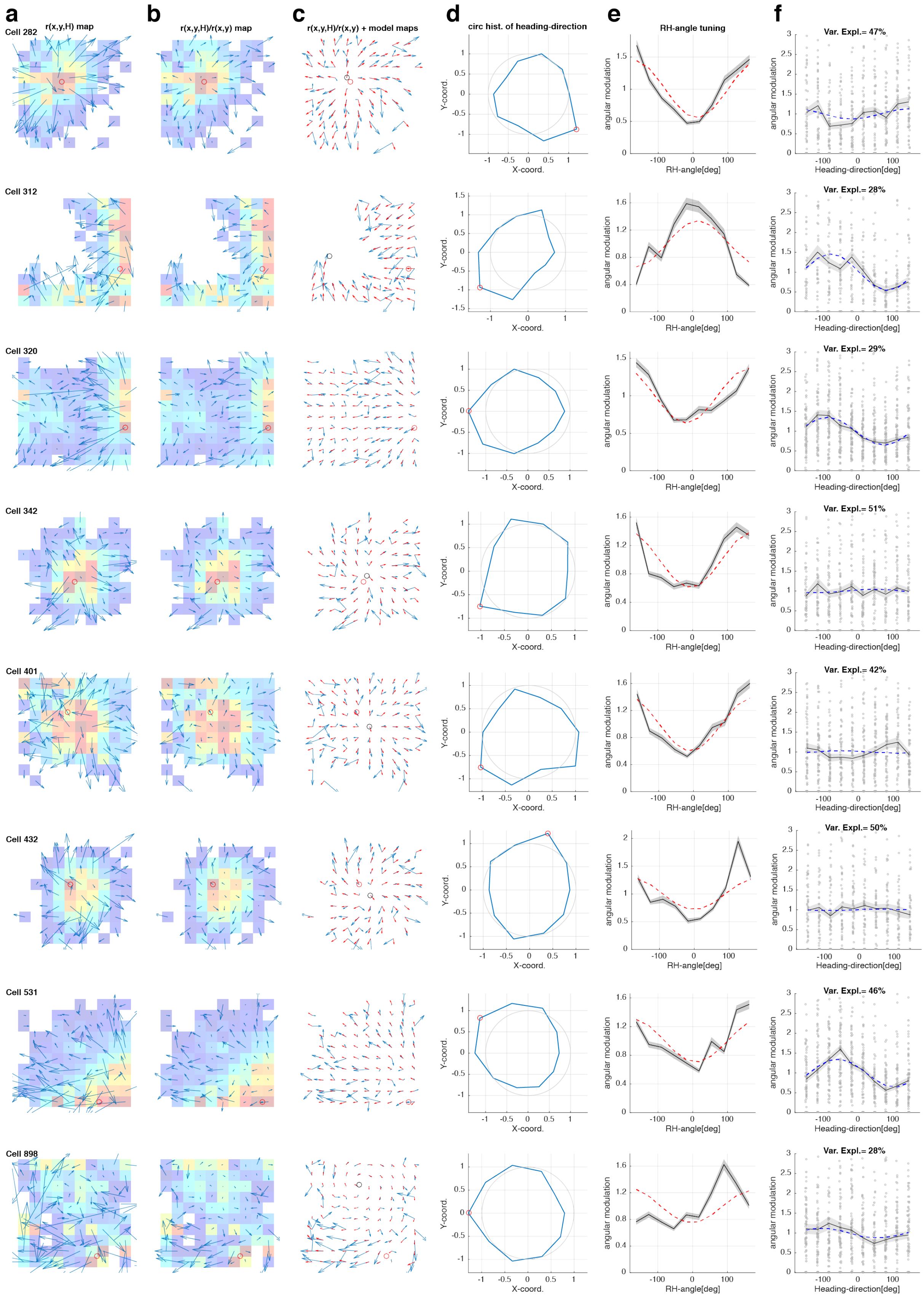

Supplementary Figure 6. a) Spatial and angular distributions of firing rates r(x,y,H) (as in Fig1c). Heat map are the representation of the spatial modulation of firing rate, from blue to red, low to high firing rate per spatial bin. Blue arrow represented the preferred direction of heading direction

response for each spatial bin. **b)** Angular distribution after spatial dependence removal (r(x,y,H)/r(x,y)). Same example cells as in a, showing spatial tuning (heat map) and heading-direction tuning (after spatial tuning subtraction) with blue arrows. **c)** Angular distribution of preferred heading direction tuning as in b (blue arrows) compared with the tuning generated by the fitted model for each neuron (red arrows). Black circles are the reference points obtained by the model. If the reference point is far from the arena enclosure, it is not drawn on the figure.
d) Heading-direction tuning averaged over the full range of +/- 180 degrees divided into 10 bins. The eccentricity (around the circle of mean rate) of the polar plot is porportional to the firing rate for the corresponding heading direction. **e)** RH-angle tuning responses relative to the reference point fitted by the model. Shaded area is the +/- st.e.m. These tunings are comparable to reports in bats (Sarel et al 2017). **f)** The RH-angle model fits (blue dashed line) and the heading-direction samples for all spatial bins and each angular bin (gray dots). Black line is the heading-direction tuning relative to the reference point fitted by the model (shaded area is the +/- st.e.m).

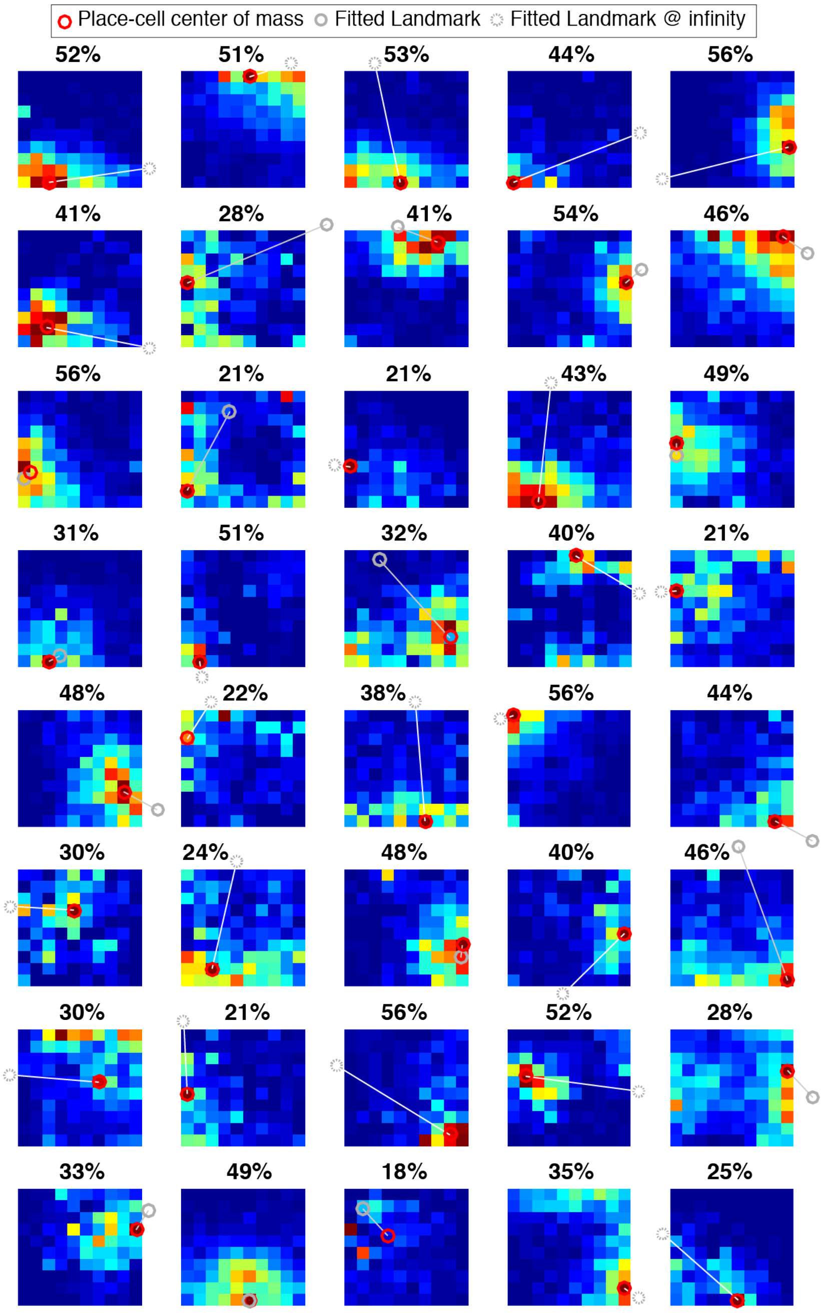

Supplementary Figure 7: Relationship between reference points and centers of mass of place fields. Red circles represent the center of mass of the place tuning map. Gray circle is the reference point when it falls within or in the proximity of the arena as shown in Fig. 3c. Dashed gray circles displaying the direction to reference point that are located at distal points from the arena (as shown in Fig. 3d,f).

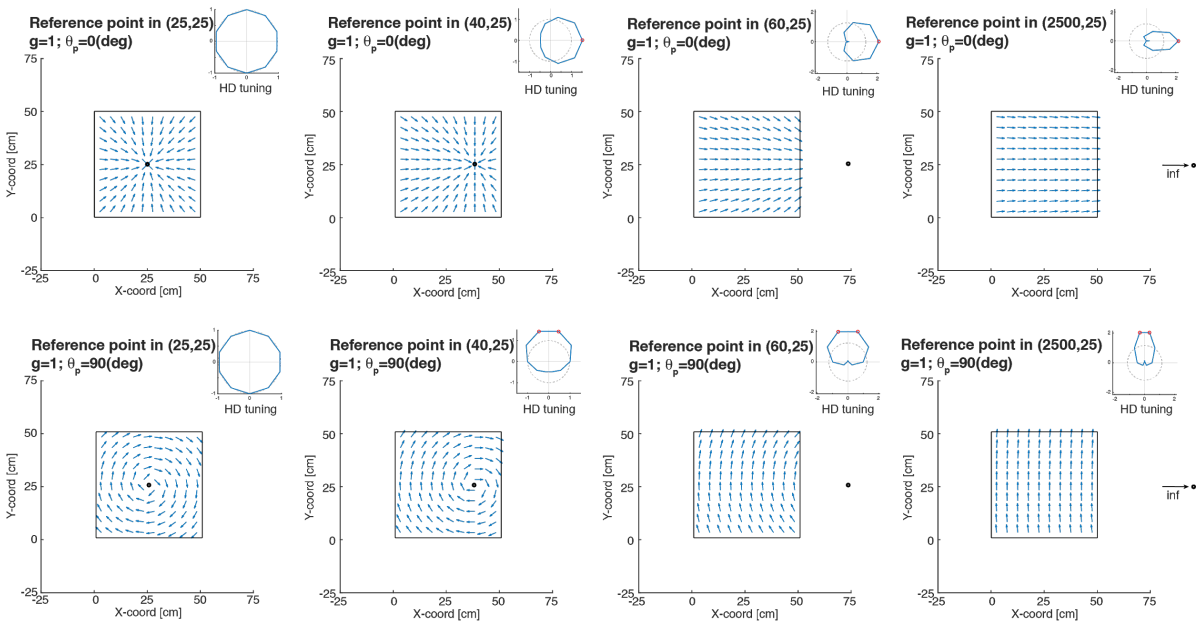

Supplementary Figure 8: Examples preferred tuning vetors generated by the model with different parameters. (Top-left) If the reference point is within the arena and the phase angle is zero (θp=0), neural response relative to the heading-direction is maximal when animal faces the reference point. **(Top-from-left to-right)** If the reference point moves away from the center of the arena, the heading-direction vector field tends to point to the reference point, but becomes a parallel field when the reference point is at infinity. The heading-direction circular histogram shows evolution towards a traditional head-direction neuronal response. **(Bottom-from-left to-right)** as on the top row, heading-direction responses behave as traditional head-direction cells when the reference is at infinity. In these examples the θp is equal to 90°, so the maximal neural response occurs when the animal moves perpendicular to a line toward the reference point.

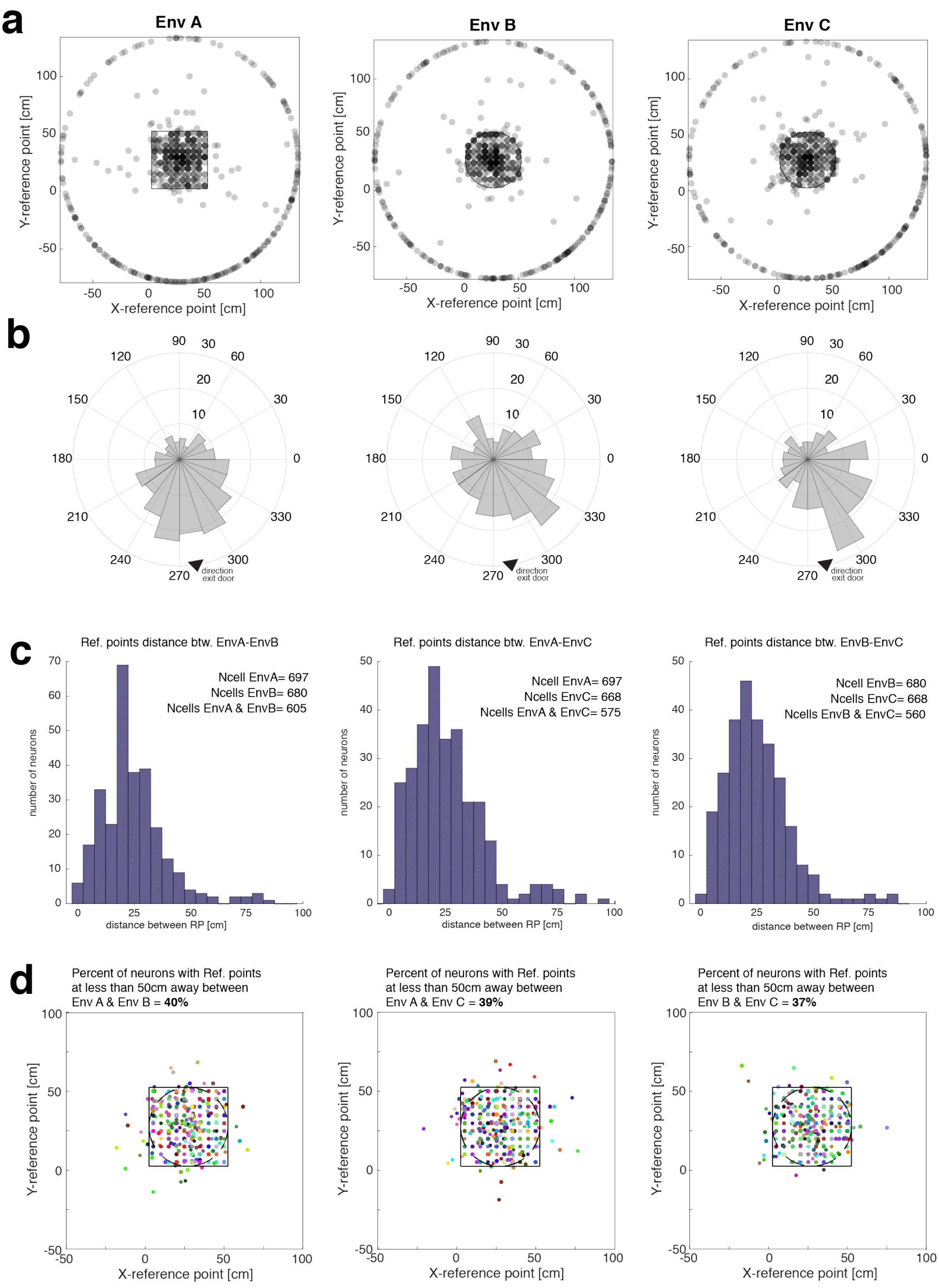

Supplementary Figure 9: Distribution of reference points for different environments (arenas). The same neurons were recorded for animals in three different environments on the same day for about 40 min in each environment (Env. A, B and C). The environments have distinct shapes, as well as wall and floor colors. **a)** Distribution of reference points within

the arena (inside the solid line square or circle), at the proximity (outside the shape with solid line), and at distal locations, collapsed to a circle around the arena. Distributions of reference points within and in the proximity of the arena do not show any bias. **b)** Distribution of reference point at distal locations displays a concentration toward the south-east direction of the room for all three arenas. This direction coincides with the exit door of the experimental room (black arrowhead pointing south direction). **c)** Distance between reference points of the same neuron in different environments. Place-field locations are completely random between environments, corresponding to global remapping. **d)** Only a subset of neurons (~40%) have reference point less than 50 cm separated between any two environments. Points of the same color show reference points for a single neuron in two environments.
